# Bilateral congenital foot amputations from amniotic band syndrome

**DOI:** 10.11604/pamj.2025.52.76.49493

**Published:** 2025-10-17

**Authors:** Fatima Zahraa Belhaj, Mohamed Sellouti

**Affiliations:** 1 Children's Hospital, Ibn Sina University Hospital Centre, Rabat, Morocco,; 2 Hôpital Militaire d'Instruction Mohammed V, Université Mohammed V, Rabat, Maroc

**Keywords:** Amniotic band syndrome, congenital amputations, congenital limb defects

## Image in medicine

A term female neonate weighing 2600g with an Apgar score of 10/10 was born after an unmonitored pregnancy. Clinical examination showed bilateral, symmetrical terminal absence of the feet with well-healed distal skin. There were no dysmorphic features, and the systemic malformation screen, including echocardiography, transfontanellar and renal ultrasound, was normal. The presentation was compatible with amniotic band syndrome (ABS), a disorder in which fibrous amniotic strands constrict developing limbs, leading to distal atrophy or congenital amputations. A multidisciplinary discussion involving neonatology, pediatric orthopaedics, plastic surgery, physical and rehabilitation medicine, and psychosocial support was held. Immediate management focused on stump protection, skin care, parental counselling, and early physiotherapy. Orthopaedic planning included delayed soft-tissue revision and stump shaping in late infancy, with early prosthetic fitting anticipated around 9-12 months to support standing and gait training. At the 3-month follow-up, the infant showed appropriate growth, intact stump coverage without infection, and good tolerance of physiotherapy.

**Figure 1:**
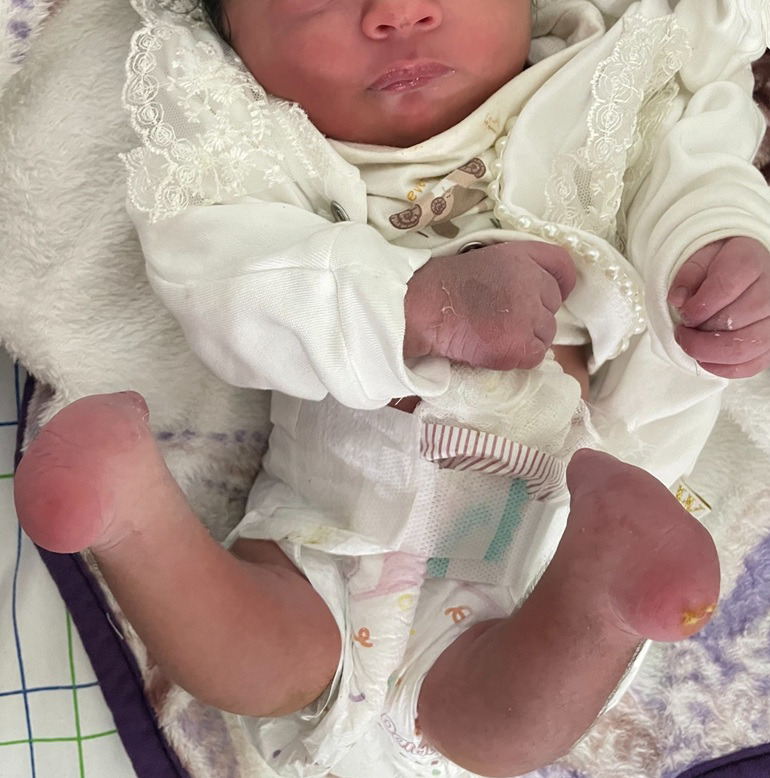
bilateral symmetrical congenital absence of the feet consistent with amniotic band syndrome

